# Outcomes of Vertebral Body Tethering in Adolescent Idiopathic Scoliosis: A Prospective, Multicenter Study

**DOI:** 10.2106/JBJS.OA.25.00309

**Published:** 2026-01-13

**Authors:** Hans K Nugraha, Lawrence L. Haber, Daniel G. Hoernschemeyer, Patrick J. Cahill, Amer F. Samdani, Firoz Miyanji, Peter O. Newton, A. Noelle Larson

**Affiliations:** 1 Department of Orthopedic Surgery, Mayo Clinic, Rochester, Minnesota; 2 Ochsner Children's Hospital, New Orleans, Los Angeles; 3 University of Missouri Health Care, Columbia, Missouri; 4 Division of Orthopedic Surgery, Children's Hospital of Philadelphia, Philadelphia, Pennsylvania; 5 Shriners Children's Philadelphia, Philadelphia, Pennsylvania; 6 British Columbia Children's Hospital, Vancouver, Canada; 7 Rady Children's Hospital, San Diego, California

## Abstract

**Background:**

Vertebral body tethering (VBT) for adolescent idiopathic scoliosis (AIS) is an alternative to posterior fusion. There are limited prospective, multicenter data available on VBT following US Food and Drug Administration approval. We hypothesize that curve correction on first postoperative standing (first erect, FE) imaging is associated with higher rates of successful correction at final follow-up.

**Methods:**

All qualifying patients with AIS who underwent thoracic and lumbar VBT between 2019 and 2022 were prospectively enrolled from 9 institutions. Radiographic and clinical data were compared preoperatively, at FE, and at final follow-up with minimum of 2 years. Success was defined as major curve magnitude of ≤35° at final follow-up and no fusion surgery.

**Results:**

One hundred twenty-seven patients were enrolled (79.5% female), with mean follow-up 2.4 years. Mean age at surgery was 12.9 ± 1.4 years, most had bone age of Sanders 4 or lower (93/112, 83.0%). In average, 7.6 ± 1.7 levels were tethered. Mean preoperative major curve magnitude was 50 ± 8°, with mean initial correction at FE of 29 ± 8° (% correction, 39 ± 18%). At final follow-up, mean curve magnitude was maintained at 26 ± 11° (% correction, 45 ± 23%) despite 29% of tether breakage. Patients who had mean FE curve magnitude of ≤35° were 88% successful compared with only 60% in those with >35° on FE (p = 0.0021). Patients showed stable sagittal alignment across all timepoints. Scoliosis Research Society-22 scores improved significantly by 2 years (p < 0.0001).

**Conclusion:**

This was the first prospective, multicenter study to assess outcomes of VBT for patients with AIS. VBT shows promise, but optimal results may depend on careful patient selection and surgical technique. FE major curve magnitude of ≤35° was associated with 88% success rate compared with only 60% success for those with poor correction.

**Level of Evidence:**

Level II. See Instructions for Authors for a complete description of levels of evidence.

## Introduction

Adolescent idiopathic scoliosis (AIS) is the most prevalent spinal deformity in children^1-4^. Despite less predictable correction and higher revision rates compared with posterior spinal fusion (PSF)^5^, vertebral body tethering (VBT) has become popular to correct AIS deformity in skeletally immature patients^6^ as it preserves motion of the instrumented thoracic^7^ and lumbar^8^ vertebrae.

According to Heuter-Volkman principle, spinal growth can result in correction of spinal deformity^9-12^. VBT's popularity among patients and parents likely reflects its potential to preserve spinal motion, a key consideration in shared decision-making for skeletally immature patients with AIS, though further studies are needed to quantify functional benefits^13^. There are limited postapproval, prospective, multicenter data available on this procedure following US Food and Drug Administration Approval in 2019 for skeletally immature AIS patients with thoracic and/or lumbar curves of 30 to 65°^14^.

The purpose of this study was to evaluate clinical outcomes and complications of VBT for AIS from prospectively collected, multicenter database. We hypothesize that patients presenting with major curve magnitude of ≤ 35° at first-erect (FE) radiograph will demonstrate higher success rates. Furthermore, we anticipate that multicenter data may provide more reliable information on clinical results of VBT across multiple institutions.

## Methods

Patients with AIS undergoing VBT were consecutively and prospectively enrolled between 2019 and 2022 at 9 tertiary-level institutions across North America. IRB approval was obtained at each center, and parents/guardian were consented for study before surgery (IRB# 19-010211). Per US FDA indications, inclusion criteria were skeletal immaturity, defined as Sanders stage ≤4 or Risser 0 to 2, flexible curve, and curve magnitudes between 30 and 65°. Curve flexibility was assessed via bending radiographs with standard AIS protocols (supine or standing lateral bends). Treatment decisions followed standard clinical practice, with VBT offered to eligible patients after discussions including PSF as an alternative, per FDA HDE criteria.

Patient demographics and perioperative data were collected, including number of levels tethered, length of stay, intraoperative complications, and postoperative complications. VBT was performed by 2 to 3 surgeons per center, with case distribution varying by center volume. Access surgeons were employed for both thoracic and lumbar approaches per standard practice. Tether configuration decisions were based on surgeon assessment of curve magnitude, flexibility, and growth potential. Double-row tethers involved 2 parallel tethers on convex side for enhanced correction in stiffer curves, while bilateral tethers addressed both major and compensatory curves. Thoracic VBT was typically performed thoracoscopically to minimize invasiveness, while lumbar VBT might use open or miniopen approach. Study timepoints included preoperative visits, at 6 month, 1 year, 2 years, and at most recent follow-up. The FE radiograph was obtained at first postoperative follow-up, typically within 2 to 4 weeks, per standard AIS practice. Centralized radiographic measurements were performed by trained analysts^15^. Skeletal maturity was assessed using the Sanders skeletal maturity staging system via hand radiographs^16^ and the Risser classification system from pelvic radiographs^17^. Suspected tether breakage was identified by change of ≥6° between screws on consecutive standing spine radiographs^18,19^. In accordance with current literature, success was defined as major curve magnitude of ≤35° at latest follow-up without conversion to PSF^12,20,21^.

Statistical analyses were performed using BlueSkyStatistics10.3.4 (BlueSky Statistics LLC) and GraphPadPrism10.4.2 (GraphPad Software Inc.). Continuous variables are reported as means with standard deviations and ranges. The Fisher exact test was used to compare success and failure based on major curve magnitude of ≤35° or >35° at FE. Analysis of variance tests were used to assess differences in total height, sagittal parameters (lumbar lordosis, T2-T12 kyphosis, and T5-T12 kyphosis), and average Scoliosis Research Society (SRS)-22 scores^22^. Spearman tests were used to assess possible correlation between bending thoracic curve magnitude at preoperative and at FE, as well as between bending lumbar curve magnitude at preoperative and at FE. Logistic regression was performed to evaluate association between success and FE major curve magnitude, adjusting for patient age at surgery, Risser stage, Sanders hand bone age, preoperative major curve magnitude, body mass index (BMI), and time to latest follow-up. Similarly, linear regression was performed to assess relationship between FE major curve magnitude and final major curve magnitude. Statistical significance was set at α ≤0.05.

## Results

One hundred twenty-seven patients were enrolled (79.5% female), with minimum 2-year and mean 2.4-year follow-up. Mean age at surgery was 12.9 ± 1.4 years. Most had hand bone age of Sanders 4 or lower (93/112, 83.0%). Lenke 1 curves were the most common (58.4%), followed by Lenke 6 (13.4%). On average, 7.6 ± 1.7 levels were tethered, with 23 patients (18%) having bilateral and 19 patients (15%) having with double-row tethers.

Mean operative time was 247 min (range 129-482 min), with 93 mL of EBL (range 50-350 mL). Average length of stay was 3.4 days. Preoperative mean major curve was 50 ± 8°, with mean initial correction at first standing of 29 ± 8° (% correction, 39 ± 18%). At 2-year follow-up, mean major curve magnitude was maintained at 26 ± 11° (45 ± 23% correction). There was almost no difference between successful and unsuccessful groups in terms of demographics and characteristics (Table I).

**TABLE I:** Comparison of Patient Demographics and Characteristics by FE Curve Magnitude

	Successful at FE (n = 112)	Unsuccessful at FE (n = 15)	p	Overall (n = 127)
Age at surgery	12.9 ± 1.3	13.0 ± 1.6	0.658	12.9 ± 1.4
Maximum preop curve	48.5 ± 7.9	53.2 ± 8.1	**0.00** **5** *	49.3 ± 8.1
Maximum preop bending curve	27.4 ± 10.6	36.1 ± 10.2	**0.003** *	29.3 ± 11.0
Height (cm)	156.9 ± 9.9	159.2 ± 9.5	0.368	157.2 ± 9.9
Weight (kg)	47.4 ± 12.3	49.7 ± 8.1	0.449	47.7 ± 11.8
BMI	19.1 ± 3.7	19.3 ± 4.1	0.748	19.2 ± 3.9
Risser	1.2 ± 1.2	1.0 ± 1.4	0.579	1.1 ± 2.8
Sanders	4.2 ± 1.7	3.8 ± 1.9	0.257	3.8 ± 1.4
Time to latest follow-up	2.4 ± 0.4	2.5 ± 0.5	0.210	2.4 ± 0.4

* Bold indicates statistical significance. BMI = body mass index, and FE = first erect.

**Fig. 1:**
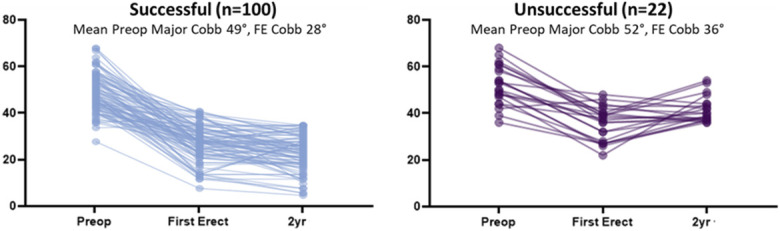
Comparison of major curve magnitude at final follow-up between patients who had a mean FE major curve ≤35° and those with >35° on FE. Five patients who underwent fusion were excluded from the analysis. FE = first erect.

With success defined as no fusion and curve<35° at latest follow-up, 95 of 127 patients had FE major curve ≤35°(mean 12.9 years), with 87 (91.6%) successful at latest follow-up. Thirty-two had FE >35°(mean 13 years), with 13 (40.6%) successful. With same success criteria, 35 patients who had FE ≤25°(mean 13.1 years) were 97.1% successful. Excluding fusion cases, the Fisher exact test showed that patients who had mean FE major curve ≤35° were 88% successful at final follow-up, compared with only 60% success in those with >35° on FE (p = 0.0021) (Fig. 1).

**Fig. 2:**
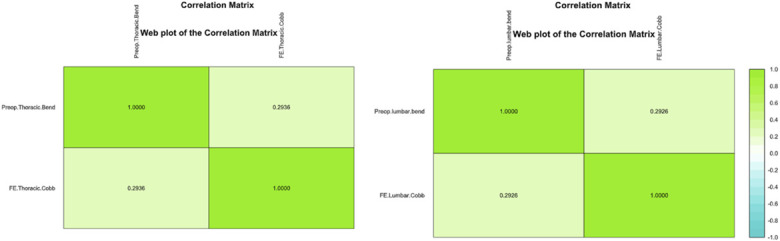
Correlation matrix between bending curve magnitude at preoperative and curve magnitude at FE. FE = first erect.

The Shapiro-Wilk test showed normal distribution of thoracic and lumbar curve magnitudes at all timepoints, as well as preoperative thoracic (w = 0.9853, p = 0.50) and lumbar bends (w = 0.9858, p = 0.26). Spearman tests showed significant, positive correlation between bending preoperative thoracic curve magnitude and FE (ρ = 0.294, p = 0.011), as well as significant positive correlation between preoperative bending lumbar curve magnitude and FE (ρ = 0.293, p = 0.019) (Fig. 2).

Controlling for age at surgery, Risser stage, Sanders hand bone age, preoperative major curve magnitude, BMI, and time-to-latest follow-up, regression analysis showed that the FE major curve is significantly associated with success at final follow-up (p < 0.001, adjusted R^2^ = 0.30). For every 1° increase in FE major curve, the final major curve increases by 0.8° (p < 0.001, adjusted R^2^ = 0.44) (Supplementary Fig. 1). Similarly, FE curve magnitude is associated with success at final follow-up (p < 0.001, adjusted R^2^ = 0.30).

By final follow-up, suspected tether breakage was most frequently noted around the thoracolumbar junction: T11-T12 (13 cases), T12-L1 (11), and T10-T11 (9). It was less frequent at T5-T6, T7-T8, L1-L2 (1 each), T8-T9 (3), and T9-T10 (5), and none at T4-T5, T6-T7, or L3-L4. (Supplementary Fig. 2).

Four patients (3%) had major complications (CSF leak, hemothorax/pleural effusions requiring sedated thoracentesis). Fifteen (11.8%) required reoperations due to overcorrections (n = 8, 6.3%), screw pullout (n = 1, 0.8%), and cord breakage (n = 1, 0.8%). Of 15 reoperations, 5 (3.9%) eventually converted to PSF. One had overcorrection resulting in fusion, and 4 had undercorrections with curve progression after VBT surgery.

There are significant differences in average SRS-22 scores across all timepoints (f = 16.56, p < 0.0001). Largest improvement occurred between preoperative and final (p < 0.001), followed by between FE and 2 years (p = 0.012), and between FE and preoperative (p = 0.021) (Table II). Sagittal parameters (T2-T12 kyphosis [f = 2.22, p = 0.11], T5-T12 kyphosis [f = 1.98, p = 0.14], lumbar lordosis [f = 0.45, p = 0.64]) were stable across all timepoints (Supplementary Table 1, Supplementary Fig. 3). Patients continued to grow postoperatively (f = 7.10, p = 0.001) (Supplementary Fig. 4).

**TABLE II:** Average Sum of SRS-22 Score Comparisons

	x̄ Difference	p
Preop SRS		
2-year SRS	0.46	**<0.001** *
Preop SRS		
FE SRS	0.22	**0.021** *
FE SRS		
2-year SRS	0.24	**0.012** *

* Bold indicates statistical significance. FE = first erect, and SRS = Scoliosis Research Society.

## Discussion

VBT's role complements PSF by preserving motion, at the cost of less curve correction. Compared with PSF, VBT offers motion preservation for select patients, though PSF provides predictable and maximal curve correction. This was the largest multicenter, prospective cohort study to date evaluating outcomes of VBT for AIS following FDA approval in 2019. VBT seemed to offer viable alternative to PSF with mean curve correction of 45 ± 23% maintained at minimum 2-year follow-up. However, success appears to hinge on the degree of correction achieved intraoperatively, with curve magnitude of ≤35° at FE strongly associated with an 88% success rate compared with 60% success in patients with FE curves >35°(p = 0.0021). Interestingly, 34 of 35 patients (97%) who had their FE at <25° achieved success. These underscore the importance of patient selection and surgical technique to optimize outcomes for this motion-preserving procedure, as the amount of correction seen overtime in most instances is relatively modest. VBT thus may act more as an internal brace rather than a true growth modulation device.

After controlling for potential confounders, FE curve magnitude remained a significant predictor of both success (p < 0.001, adjusted R^2^ = 0.30) and final curve magnitude (p < 0.001, adjusted R^2^ = 0.44). For every 1° increase in FE curve magnitude, final curve magnitude increased by 0.8°—suggesting direct relationship between early-postoperative alignment and final correction.

Unlike previous single-center study conducted before FDA approval in 2019^23^, there were no differences between the successful and unsuccessful groups in terms of body weight and BMI. Despite no differences between successful and unsuccessful groups in demographics and time-to-latest follow-up, the successful group exhibited significantly smaller preoperative major curves (48.5 ± 7.9° vs. 53.2 ± 8.1°, p = 0.005). This suggests that preoperative curve magnitude is also key determinant of achieving adequate correction. This does not intend to suggest that VBT is only for curves under 50°, as other factors such as skeletal maturity and curve flexibility also have significant impact. While curve magnitude is always a consideration, it is essential to be cognizant that these factors play additional critical role in determining whether VBT is an appropriate treatment option for patients with AIS.

Spearman tests revealed significant positive correlations between preoperative bending thoracic curve magnitude and FE (ρ = 0.294, p = 0.011), as well as between preoperative bending lumbar curve magnitude and FE (ρ = 0.293, p = 0.019). Similar to vertebral body stapling^24,25^, these emphasize the role of curve flexibility in postoperative outcomes. Patients with more flexible curves preoperatively are better positioned to achieve optimal correction at FE. Previous study has showed that FE is 10° more than intraoperative correction on the table^26^. Thus, the intraoperative correction can be used to predict correction on FE imaging.

Patients continued to grow postoperatively. Although this reflected total height, this is similar from previous study^27^ showing growth at T1-T12 at 2 years postoperatively. Sagittal balance remained consistent across time points. This reinforced VBT's capacity to maintain spinal sagittal alignment^11^, as significant alterations could predispose patients to sagittal imbalance or secondary complications^28^.

Suspected tether breakage occurred in 29% of patients—lower than previous retrospective multicenter study showing 50% by 36 months postoperatively^29^. Most occurred around thoracolumbar junction (T11-T12: 13 cases; T12-L1: 11 cases; T10-T11: 9 cases), likely reflects heightened mechanical stress in the transitional thoracolumbar zone due to increased mobility or load bearing. Nevertheless, it is notable that curve correction was maintained (26 ± 11°) at final follow-up (minimum 2 years, mean 2.4 years). This suggests that while tether integrity is important, breakage does not universally compromise outcomes, particularly if adequate correction is achieved early. One possible explanation is that remodeling and soft tissue adaptation may help maintain alignment even in the setting of tether breakage. Previous study showed that only 15% of tether breakage is significant enough to warrant reoperation^18^. In this cohort, only 1 patient required reoperation attributed to cord breakage, with overcorrection (6.3%) and screw pullout (0.8%) being more common. Future studies should explore optimal tether configuration and tensioning strategies to minimize break while maximizing correction.

The success rate of 88% in patients achieving FE curve ≤35° is comparable with historical data on VBT, where success rates typically range from 70% to 85% depending on the definition of success and follow-up duration^20,21^. Patients with larger residual curves at FE may represent subgroups with more rigid, higher preoperative curve magnitudes or suboptimal intraoperative correction. Although demographic and maturity did not differ significantly between groups (Table I), curve magnitude at preoperative may be a factor in achieving adequate correction. Surgeons might consider extending tethering levels to improve outcomes in these higher-risk patients, or starting with curves ≤50° while starting VBT practice. This may help offering more fixation points, increasing spinal alignment control, and improving deformity correction over larger spinal segment.

**Fig. 3:**
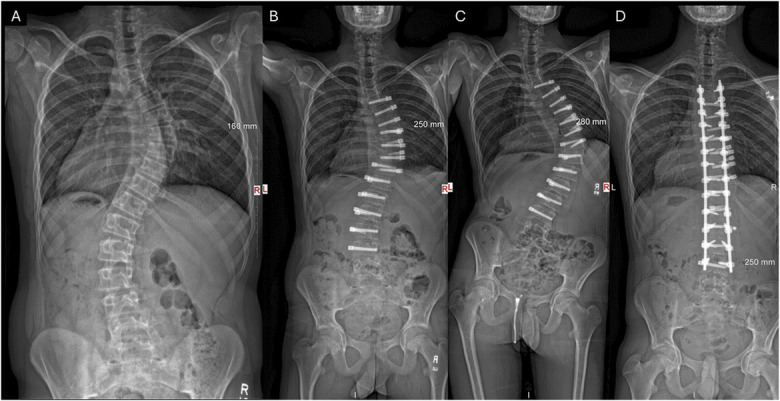
Overcorrection of a 16-year-old boy, resulting in fusion. His preoperative curve magnitude was 58° (**Fig. 3-A**) and corrected to 39° at his FE using bilateral tether and bilateral row (**Fig. 3-B**). Nevertheless, the overcorrection found at his 18-month follow-up (**Fig. 3-C**) warranted a posterior spinal fusion (**Fig. 3-D**).

Complications in this cohort were relatively low, with major complications occurring in 3% patients and 11.8% reoperations. These are consistent with prior reports on VBT, which showed 10% to 15% revision rates^5^ and 6.7% thoracic complications^30^. Notably, only 3.9% of patients converted to PSF, lower than earlier studies (5%-10%)^31^. Overcorrection, the most common reason for reoperation (6.3%) (Fig. 3) underscores the challenge of balancing correction with growth modulation and echoes ongoing debates about optimal timing for VBT^31-33^ particularly in younger patients with significant growth potential^13^.

Quality of life, assessed via SRS-22 scores, improved significantly—with greatest improvement between preoperative and 2 years (mean difference = 0.46, p < 0.001). This is higher than minimal clinically important difference of 0.4 in average sum score^34^. VBT thus not only corrects deformity but also enhances patient-reported outcomes. To our knowledge, this is also the first study to assess such functional outcomes in patients with AIS undergoing VBT.

Limitations of this study include its relatively short follow-up (minimum 2 years, mean 2.4 years). VBT's motion-preserving potential is supported by prior studies^7,8,35,36^; thus, we did not directly measure spinal ROM. Future study quantifying ROM to validate VBT's might still be needed to quantify functional benefits beyond the validated SRS-22 scores, particularly in thoracic where motion needs for activity of daily livings are less clear^37^. Potential variability in bending radiograph protocols^38^ across centers may affect flexibility assessments. Given that the majority of patients presented with thoracic curves (58.4% Lenke 1, followed by Lenke 6 (13.4%), and only a small subset (18%, n = 23) had bilateral tethers, a subgroup analysis comparing thoracic and lumbar tether outcomes, as well as sagittal parameters, was not conducted. In addition, while prospective and multicenter, the study lacks control group undergoing PSF. As trunk height was not consistently available in our multicenter registry, total height was used as a surrogate marker of growth. Variability in surgical technique across 9 institutions may also introduce heterogeneity, though this reflects real-world practice and enhances generalizability. The success definition (≤35° without PSF) is rather arbitrary but aligns with standards from previous established literature^12,20,21,25^. Although it does not specific address motion preservation, the SRS-22 score is well-validated and widely accepted; thus, improvements in the score suggest enhanced quality of life.

In conclusion, this study provides critical insights into the efficacy and challenges of VBT for AIS. Achieving a FE curve magnitude of ≤35° appears to be a pivotal determinant of success. VBT offers a motion-preserving alternative to PSF, although its outcomes are less predictable and long-term durability remains indeterminate. Head-to-head studies comparing VBT with selective fusion, particularly in terms of long-term curve control, complications, motion preservation, and quality of life, are needed to make shared decision-making even better. These could guide surgeons in patient selection and optimizing intraoperative strategies to maximize early correction. Further longitudinal studies are warranted to assess outcomes beyond 2 years and fully elucidate the risk benefit profile of VBT.

## Appendix

Supporting material provided by the authors is posted with the online version of this article as a data supplement at jbjs.org (http://links.lww.com/JBJSOA/B73). This content was not copyedited or verified by JBJS.

## APPENDIX. Other Harms Study Group Investigators

Other Harms Study Group Investigators: Aaron Buckland, MD, Melbourne Orthopaedic Group & Royal Childrens Hospital; Ahmet Alanay, MD, Acibadem Maslak Hospital, Turkey; Amit Jain, MD, Johns Hopkins Hospital, Baltimore; Baron Lonner, MD, Mount Sinai Hospital, New York; Benjamin Roye, MD, Columbia University, New York; Burt Yaszay, MD, Seattle Children’s Hospital; Caglar Yilgor, MD, Acibadem Maslak Hospital, Turkey; Daniel Hedequist, MD, Boston Children’s Hospital; Daniel Sucato, MD, Texas Scottish Rite Hospital, Dallas; David Clements, MD, Cooper Bone & Joint Institute New Jersey; Harry Shufflebarger, MD, Paley Orthopedic & Spine Institute, Palm Beach; Jack Flynn, MD, Children’s Hospital of Philadelphia; Jean Marc Mac Thiong, MD, CHU Sainte-Justine, Montreal; Josh Murphy, MD, Children's Healthcare of Atlanta; Joshua Pahys, MD, Shriners Children’s Philadelphia; Keith Bachmann, MD, University of Virginia; Kevin Neal, MD, Nemours Children’s Clinic, Jacksonville; Laurel Blakemore, MD, Pediatric Specialists of Virginia/Children’s National; Lawrence Lenke, MD, Columbia University, New York; Mark Abel, MD, University of Virginia; Mark Erickson, MD, Children’s Hospital, Denver Colorado; Michael Glotzbecker, MD, Rainbow Children’s Hospital, Cleveland; Michael Kelly, MD, Rady Children’s Hospital, San Diego; Michael Vitale, MD, Columbia University, New York; Michelle Marks, PT, MA, Setting Scoliosis Straight Foundation, San Diego; Munish Gupta, MD, Washington University, St. Louis; Nicholas Fletcher, MD, Emory University, Atlanta; Paul Sponseller, MD, Johns Hopkins Hospital, Baltimore; Peter Gabos, MD, Nemours/Alfred I. duPont Hospital for Children, Delaware; Peter Sturm, MD, Cincinnati Children’s Hospital, Betz; Randal Betz, MD, Institute for Spine & Scoliosis, New Jersey; Robert H. Cho, MD, Shriners Children’s Southern California; Stefan Parent, MD, CHU Sainte-Justine, Montreal; Stephen George, MD, Nicklaus Children's Hospital, Miami; Steven Hwang, MD, Shriners Children’s Philadelphia; Suken Shah, MD, Nemours/Alfred I. duPont Hospital for Children, Delaware; Sumeet Garg, MD, Children’s Hospital, Denver, Colorado; Tom Errico, MD, Nicklaus Children's Hospital, Miami; Vidyadhar Upasani, MD, Rady Children’s Hospital, San Diego.
